# Cost-Effectiveness of Contraceptive Use in Indonesia after the Implementation of the National Health Insurance System

**DOI:** 10.1155/2021/3453291

**Published:** 2021-05-08

**Authors:** Auliya A. Suwantika, Neily Zakiyah, Irma M. Puspitasari, Rizky Abdulah

**Affiliations:** ^1^Department of Pharmacology and Clinical Pharmacy, Faculty of Pharmacy, Universitas Padjadjaran, Indonesia; ^2^Center of Excellence in Higher Education for Pharmaceutical Care Innovation, Universitas Padjadjaran, Indonesia; ^3^Center for Health Technology Assessment, Faculty of Pharmacy, Universitas Padjadjaran, Indonesia

## Abstract

Since 2014, Indonesia has initiated to implement a national health insurance system, which included both of short- (SARC) and long-acting reversible contraceptive (LARC) into the benefit package. The aim of this study was to analyze the cost-effectiveness of contraceptive use in Indonesia after the implementation of the national health insurance in 2014-2017. A decision tree model was developed to analyze the cost-effectiveness of contraceptive use in Indonesia in 2014-2017 by comparing two strategies of pregnancy prevention: contraceptive and non-contraceptive. For contraceptive strategy, we took into account SARC and LARC. In a comparison with non-contraceptive, we calculated that the incremental cost-effectiveness ratio (ICER) of SARC would be $5.18, $4.80 and $3.76 per pregnancy averted for injection, condom, and pill, respectively. For LARC, we calculated that the ICER would be $1.67 and $0.84 for implant and intrauterine device (IUD), respectively, compared with non-contraceptive. In general, the cost-effectiveness value of LARC ($1.25) was much better than SARC ($4.58). The cost of contraceptive was considered to be the most influential parameter affecting both the ICER of SARC and LARC. In conclusion, the use of LARC in Indonesia was considered to be more cost-effective than SARC since the implementation of national health insurance system. In particular, IUD yielded the greatest cost-effectiveness value, compared with other methods.

## 1. Introduction

As one of most populous countries in the world with 268 million people, the population of Indonesia is projected to be 306 million by 2035 [1]. Despite the global increase in family planning in the last two decades, the unmet need for family planning remains a relevant issue in many countries, including in Indonesia [2]. In particular, it has been reported that one woman dies every six hours in Indonesia due to pregnancy or natal issues [3]. This high maternal mortality rate should be of high importance to Indonesian women when considering the significance of their reproductive health. Factors to be considered is family planning by modern contraceptives, including short- (SARC) and long-acting reversible contraceptive (LARC). Unfortunately, the contraceptive prevalence rate in Indonesia improved only marginally from 57.4% in 1997 to 61.9% in 2012, and the country has the highest maternal mortality rate in Southeast Asia, of 359 per 100,000 live births [4]. However, family planning by modern contraceptives is crucial to improve maternal health and to reduce the number of maternal deaths [5].

Over the last 10 years, many efforts to increase women's access to modern contraceptives in Indonesia have been implemented by addressing issues related to policy, financing, supply, service delivery, and sociocultural [3]. Since 2014, the country has initiated to implement a national health insurance system, which included both of SARC and LARC into the benefit package. A recent study reported that scaling up access to modern contraceptive could improve women's health outcomes substantially and be cost-effective or even cost saving across a range of scenarios compared to the current situation [6]. However, this evidence in Indonesia is supported by a limited number of studies. Therefore, further studies are needed to generate more comprehensive economic evaluations on supporting evidence-based policy related to family planning by using modern contraceptives. This study is aimed at analyzing the cost-effectiveness of contraceptive use in Indonesia after the implementation of the national health insurance in 2014-2017.

## 2. Methods

**Figure 1:**
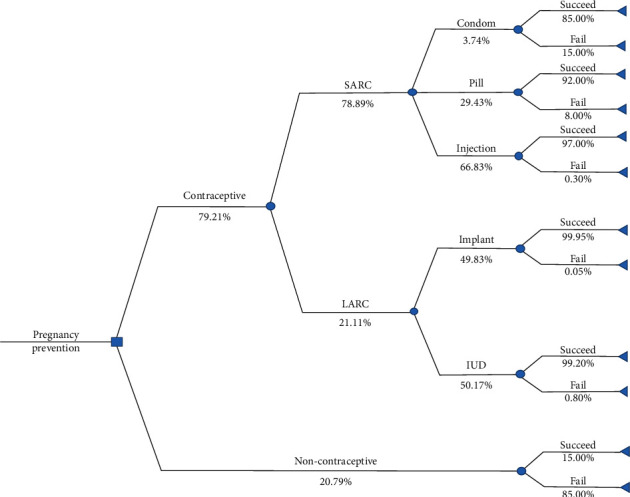
Decision tree model.

We developed a decision tree model to analyze the cost-effectiveness of contraceptive use in Indonesia during a period of 2014-2017 by comparing two strategies of pregnancy prevention: the use of contraceptive and non-contraceptive. For contraceptive, we took into account SARC and LARC. In accordance with data from the Indonesia Health Profile [7–10], we categorized condom, pill, and injection in SARC. Implant and intrauterine device (IUD) were categorized in LARC. We estimated the probability of using contraceptive and non-contraceptive to prevent pregnancy by considering the number of women in reproductive age (15-49 years old) and the number of family planning acceptors on the same age group. The probabilities of using contraceptive and non-contraceptive were estimated to be 79.21% and 20.79%, respectively. The probabilities of using SARC and LARC were estimated to be 78.89% and 21.11%, respectively. The same approach was applied to estimate the probabilities of using contraceptive in all specific methods (see Figure 1).

To estimate the annual cost of using contraceptive, we applied a payer perspective, which considered all costs covered by the Indonesian national health insurance system (*BPJS Kesehatan*). For LARC, the insurance would cover costs of implant and IUD at $7.08 [11]. Since implant and IUD were assumed to be used up to 5 and 10 years, respectively, we calculated that the annual costs would be $1.42 and $0.71 for these respective methods [11, 12]. We considered only non-hormonal IUD because of the insurance policy [11]. For SARC, we estimated that the annual cost of pill would be $2.89 by considering its catalogue price at $0.22, and its average use in a year would be 13 [13, 14]. We estimated that the annual cost of injection would be $4.25 by applying its catalogue price at $1.06 and considering it should be administered four times in a year [13, 15]. For condom, we specifically assumed that the average use would be 112 per year, and its cost per use would be $0.03 [12, 16]. We calculated the annual cost of condom would be $3.36 [13, 16]. Regarding the effectiveness, the potential of pregnancy averted was applied as the effectiveness on this study by considering data from the Ministry of Health on the effectiveness of contraceptive methods as commonly used in the first year [17]. For SARC, we estimated the effectiveness rates would be 85.00%; 92.00%, and 97.00% for condom, pill, and injection, respectively. For LARC, we estimated the effectiveness rates would be 99.20% and 99.95% for IUD and implant, respectively. For non-contraceptive, the effectiveness rate would be 15.00% (see Table 1) [17].

The incremental cost-effectiveness ratio (ICER) in cost per pregnancy averted was calculated by comparing the use of contraceptive with non-contraceptive. Univariate sensitivity analysis was performed to investigate the effects of different input parameters on the ICER by taking into account lower and upper values of each parameter in different scenarios. For LARC, we calculated lower and upper values of cost parameters by considering longer and shorter durations of use in a comparison with the base-case value, respectively. We applied 6 and 12 for longer durations of use in implant and IUD, respectively [12]. We also applied 3 and 8 years for shorter durations of use in the respective methods [12]. For SARC, we applied different approaches in lower and upper values. Applying the same prices, we calculated that lower and upper values of annual cost for condom by considering different average uses of condom would be 89 and 144 per year, respectively [16]. Applying the same average use in a year, we calculated lower and upper values of annual cost for pill by considering costs per use at $0.15 and $0.25, respectively [14]. Considering that injection would be administered four times in a year, we calculated lower and upper values of annual cost for injection by considering costs per use at $0.71 and $1.42, respectively [15]. To calculate lower and upper values of effectiveness parameters, we varied each parameter at the base-case value of ±5%.

## 3. Results

The numbers of SARC users were estimated to be 26.15 million, 26.68 million, 26.87 million, and 19.18 million, respectively, in 2014-2017. In particular, the number of LARC users would be 7.58 million, 7.63 million, 7.92 million, and 3.34 million in the same period. Despite the fact that total number of users in both of SARC and LARC fluctuated during 2014-2017, injection and implant methods were considered as the most commonly used contraceptives in SARC and LARC, respectively (see Table 2(a)).

The total costs of SARC use were estimated to be $98.89 million (92.54%), $100.95 million (92.58%), $101.92 million (92.31%), and $75.73 million (95.54%) in 2014-2017, respectively. In addition, the total costs of LARC would be $7.97 million (7.46%), $8.09 million (7.42%), $8.49 million (7.69%,) and $3.53 million (4.46%) in the same period. Obviously, the total costs of SARC and LARC fluctuated during 2014-2017. Furthermore, the use of SARC was much higher than LARC in the same period (see Table 2(b)).

**Figure 2:**
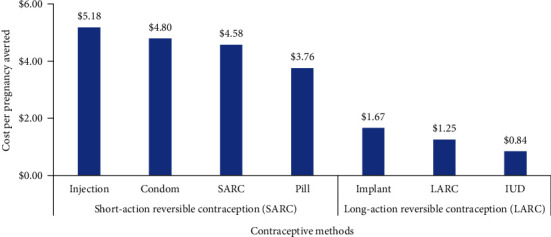
Cost-effectiveness values among contraceptive methods.

In a comparison with non-contraceptive, we calculated the ICER of SARC would be $5.18, $4.80, and $3.76 per pregnancy averted for injection, condom, and pill, respectively. Additionally, we calculated that the ICER of LARC would be $1.67 and $0.84 for implant and IUD, respectively. In general, the cost-effectiveness value of LARC ($1.25) was much better than SARC ($4.58) (see Figure 2).

**Figure 3:**
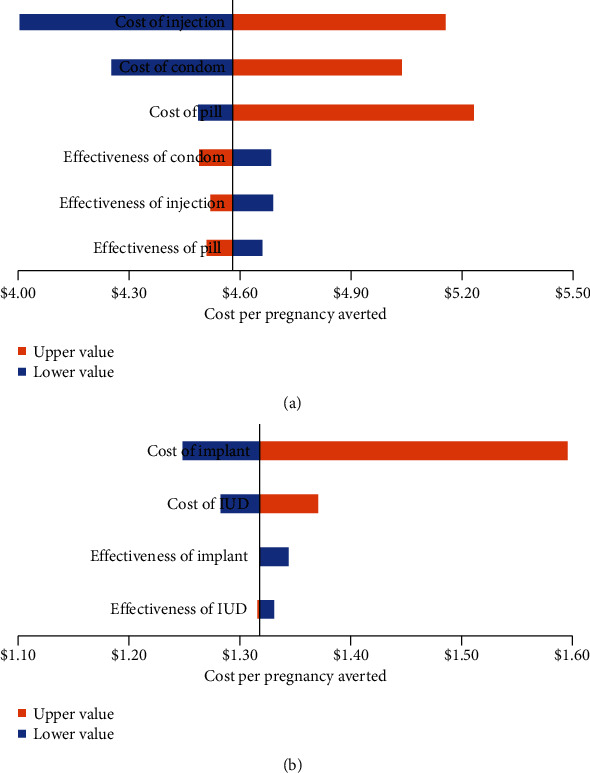
(a) One-way sensitivity analysis of SARC. (b) One-way sensitivity analysis of LARC.

In addition, sensitivity analysis explored the robustness of cost per pregnancy averted in SARC and LARC under the uncertainty by characterizing input parameters and assumptions that were utilized in the model. The result from the sensitivity analysis showed that the cost of contraceptive was considered to be the most influential parameter affecting both the ICER of SARC (see Figure 3(a)) and LARC (see Figure 3(b)).

## 4. Discussion

Despite the substantial efforts on reducing fertility rates that have been made in the last couple decades in Indonesia, significant programmatic issues remain exist and threaten the decline of the fertility rate. As one of the countries with the highest maternal mortality rate in the world, strengthening family planning by using modern contraceptive through comprehensive policy, financing, supply, service delivery, and sociocultural strategies is crucial to reduce the number of maternal deaths significantly [18–22]. Despite the fact that the use of LARC in Indonesia was calculated to be much lower than SARC, this study confirmed that the use of LARC was considered to be more cost-effective than SARC. This result reconfirmed the result from a previous study by Mavranezouli et al. about the cost-effectiveness of LARC in the UK, which highlighted that even though the uptake of LARC was considered to be low in developed countries, this method proved to be cost-effective from the British National Health Service perspective [23]. In particular, our study also mentioned that the effectiveness of LARC (99.20-99.95%) was considered to be higher than SARC (85.00-97.00%). This result is linear with the result from a study by Trussel et al., which estimated that the percentage of women experiencing an unintended pregnancy during the first year of typical use in LARC (0.05%-0.8%) was lower than SARC (6-18%) [24].

For LARC, the ICER of IUD ($0.84) showed the greatest cost-effectiveness value, compared with other methods. This finding is similar with a previous published study by Sonnenberg et al., which confirmed that contraceptive methods requiring less frequently action were less costly and more effective than methods requiring daily basis action [25]. In a comparison with non-contraceptive, IUD was reported to be one of the most cost saving methods [25]. The result of our study highlighted the potential cost-effectiveness value of IUD to be considered as the major contraceptive method in Indonesia. For SARC, pill as an oral contraceptive was considered to be the most cost-effective SARC by resulting the lowest ICER ($3.76). Another study by Foster et al. mentioned that over half of the pregnancies averted in their study were attributable to oral contraceptive with approximately $3.37 of savings for every dollar spent in using this method [26].

Our study gives some useful insight into the cost-effectiveness of contraceptive methods by making comparison between LARC and SARC, but we should highlight several limitations of this study. Firstly, we considered this study from one perspective only. We did not consider costs from other perspectives, such as patient's perspective that might be able to capture the cost of side-effects in all contraceptive methods. In a lot of cases, side-effects of contraceptive are evident. Secondly, we did not take into account the capital costs that might lead the policy makers into poor resource-allocation decisions. However, equipment costs in one contraceptive method might be much higher than other contraceptive methods. Thirdly, unlike cost, a discount rate of health outcome was not applied in this study since we assumed that health outcome in our study would be less desirable with time.

This study is not the first economic evaluation study on the contraceptive use in Indonesia. Yet, it has several major novelties. Compared to a previous study that analyzed the cost-effectiveness of scaling up modern family planning interventions in Indonesia and Uganda [6], our study has some significant differences in the process of analysis. Firstly, we focused our study specifically in Indonesia by developing a hypothetical model and taking local data into account. However, the key challenge to conduct economic evaluation studies in Indonesia is the difficulty in obtaining local data. Secondly, we focused our study on payer perspective. However, it was important to be taken into account because Indonesia has started to implement the national health insurance system since 2014, and contraceptive has been included as the benefit package. Because country situations and programmatic challenges vary so greatly in Indonesia, we hope that the result of this study can assist the stakeholder to develop guidelines on criteria for contraceptive use according to national health policies, needs, priorities, and resources, while reflecting upon local values and preferences.

## 5. Conclusion

The use of LARC in Indonesia was considered to be more cost-effective than SARC since the implementation of the national health insurance system. In particular, IUD yielded the greatest cost-effectiveness value, compared with other methods. The cost of contraceptive was considered to be the most influential parameter affecting both the ICER of SARC and LARC.

## Figures and Tables

**Table 1:** Parameter used in the decision tree model.

Parameters	Value	Reference
Epidemiology		
Probability of using SARC	75.71%	[7–10]
Probability of using condom	3.74%	[7–10]
Probability of using pill	29.43%	[7–10]
Probability of using injection	66.83%	[7–10]
Probability of using LARC	24.29%	[7–10]
Probability of using implant	41.56%	[7–10]
Probability of using IUD	41.85%	[7–10]
Probability of using non-contraceptive	20.22%	[7–10]
Annual costs		
Condom	$5.36	[13, 16]
Pill	$2.89	[13, 14]
Injection	$4.25	[11, 15]
Implant	$1.42	[11, 13]
IUD	$0.71	[11, 13]
Effectiveness		
Non-contraceptive	15.00%	[17]
Condom	85.00%	[17]
Pill	92.00%	[17]
Injection	97.00%	[17]
Implant	99.95%	[17]
IUD	99.20%	[17]

**Table:** (a) Total number of contraceptive user and pregnancy averted in 2014-2017

Methods	2014		2015		2016		2017	
	Number of users	Pregnancy averted	Number of users	Pregnancy averted	Number of users	Pregnancy averted	Number of users	Pregnancy averted
Non-contraceptive	6,910,634	1,036,595	6,051,954	907,793	6,196,135	929,420	13,732,047	2,059,807
SARC	26,145,620	24,812,992	26,683,685	25,325,011	26,866,476	25,505,859	19,175,895	18,362,519
Condom	1,110,341	943,790	1,131,373	961,667	1,171,509	995,783	288,388	245,130
Pill	8,300,362	7,636,333	8,447,972	7,772,134	8,280,823	7,618,357	4,069,844	3,744,256
Injection	16,734,917	16,232,869	17,104,340	16,591,210	17,414,144	16,891,720	14,817,663	14,373,133
LARC	7,576,897	7,543,888	7,628,305	7,595,690	7,920,260	7,887,406	3,338,912	3,324,578
Implant	3,680,816	3,678,976	3,788,149	3,786,255	4,067,699	4,065,665	1,650,227	1,649,402
IUD	3,896,081	3,864,912	3,840,156	3,809,435	3,852,561	3,821,741	1,688,685	1,675,176

**Table:** (b) Total cost of contraceptive use in 2014-2017

Methods	2014	2015	2016	2017
Non-contraceptive	$0	$0	$0	$0
SARC	$98,886,812	$100,954,929	$101,922,670	$75,728,863
Condom	$3,731,636	$3,802,321	$3,937,210	$969,215
Pill	$24,028,519	$24,455,831	$23,971,956	$11,781,694
Injection	$71,126,657	$72,696,777	$74,013,504	$62,977,954
LARC	$7,974,579	$8,087,025	$8,491,860	$3,534,136
Implant	$5,214,728	$5,366,790	$5,762,838	$2,337,929
IUD	$2,759,851	$2,720,235	$2,729,022	$1,196,207

## Data Availability

Datasets used and/or analyzed during the current study are available from the corresponding author upon reasonable request.
